# Molecular epidemiology and resistance mechanisms of carbapenem-resistant Gram-negative bacilli in a referral hospital in Lima, Peru

**DOI:** 10.1093/jacamr/dlag236

**Published:** 2026-10-21

**Authors:** Eddie Angles-Yanqui, Katherine Yauri-Condor, Jorge Velásquez-Pomar, Rosa Hernandez-Hernandez, Angel Salazar, Pamela Yañez, Diana Flores-Leon, Ronnie G Gavilan

**Affiliations:** Facultad de Medicina Alberto Hurtado, Universidad Peruana Cayetano Heredia, Lima, Perú; Centro de Investigaciones Tecnológicas, Biomédicas y Medioambientales (CITBM), Universidad Nacional Mayor de San Marcos, Lima, Perú; Servicio de Microbiología, Hospital Nacional Arzobispo Loayza, Lima, Perú; Servicio de Microbiología, Hospital Nacional Arzobispo Loayza, Lima, Perú; Servicio de Microbiología, Hospital Nacional Arzobispo Loayza, Lima, Perú; Centro Nacional de Salud Pública, Instituto Nacional de Salud, Lima, Perú; Centro Nacional de Salud Pública, Instituto Nacional de Salud, Lima, Perú; Escuela Profesional de Medicina Humana, Universidad Privada San Juan Bautista, Lima, Perú; Centro Nacional de Salud Pública, Instituto Nacional de Salud, Lima, Perú; Escuela Profesional de Medicina Humana, Universidad Privada San Juan Bautista, Lima, Perú

## Abstract

**Background:**

Carbapenem-resistant Gram-negative bacilli (CR-GNB) represent a significant public health threat, as they are associated with high mortality in healthcare settings. Carbapenemase production is the predominant resistance mechanism, and horizontal gene transfer drives the rapid dissemination of antimicrobial resistance (AMR).

**Objectives:**

This study aimed to characterize the phenotypic and genotypic AMR profiles of CR-GNB isolated from patients at Arzobispo Loayza National Hospital (Lima, Peru) between October 2021 and January 2022.

**Methods:**

We conducted a cross-sectional study and collected clinical and epidemiological data from patients with bacterial infections. MALDI-TOF identified pathogens, and antimicrobial susceptibility was interpreted according to CLSI guidelines. A subset of isolates underwent whole-genome sequencing (Illumina) to determine genomic resistance markers.

**Results:**

A total of 159 patients were included (median age = 54 years; 66.7% male); 72.3% had confirmed clinical infections, and 66% were admitted to the ICU. The most frequent infection types were pneumonia, urinary tract infection and bacteraemia. Overall, 97.5% of isolates were classified as XDR. Carbapenem resistance approached 100% (imipenem 99.4%, meropenem 98.7%). Whole-genome data revealed clonal diversity among *Klebsiella pneumoniae* (ST13/ST15) carrying *bla*_NDM-1_; *Acinetobacter baumannii* uniformly harboured *bla*_OXA-23_; and *Pseudomonas aeruginosa* included high-risk clones ST357 (*bla*_IMP-93_) and ST111 (*bla*_IMP-18_, *bla*_VIM-2_).

**Conclusions:**

These findings demonstrate a critical burden of XDR-GNB and the presence of multiple carbapenemases (NDM, IMP, KPC and VIM) associated with clinically relevant high-risk lineages. Strengthening molecular surveillance, optimizing antimicrobial stewardship and reinforcing infection-control measures are urgently needed to contain CR-GNB in critical-care settings in Peru.

## Introduction

The emergence and spread of bacteria resistant to multiple classes of antibiotics represent one of the leading threats to global public health.^1^ This phenomenon, particularly critical in low- and middle-income countries, entails clinical, social and economic consequences and, in 2021, was estimated to be associated with approximately 4.71 million deaths attributable to antimicrobial resistance (AMR).^2,3^ In this context, hospital-acquired infections provide a particularly favourable setting for the emergence and dissemination of AMR pathogens, driven by the concentration of immunocompromised patients as well as the intensive and prolonged use of broad-spectrum antimicrobial agents.^4^ This dynamic has promoted the increase of MDR and even XDR infections, significantly compromising the therapeutic management of common conditions, such as respiratory^5^ and urinary tract infections,^6^ by progressively limiting the available antimicrobial options.^7^

Given this situation, *Klebsiella pneumoniae* (46.6%), *Pseudomonas aeruginosa* (36%) and *Acinetobacter baumannii* (10.6%) rank among the most frequently isolated pathogens in hospitals.^8^ These species, members of the ESKAPE group designated by the World Health Organization as priority pathogens, are characterized by their high virulence and remarkable capacity to develop and disseminate resistance to multiple classes of antimicrobials.^4,9^ Infections caused by carbapenemase-producing Enterobacterales (CRE) have mortality rates that can reach 50.3%,^10^ reflecting the severe therapeutic challenges they pose, even in the presence of last-line antimicrobials.^11^ In light of this threat, it is essential to complement phenotypic diagnosis with genotypic tools, which enable a more precise characterization of resistance mechanisms and the surveillance of high-risk clones.

The primary resistance mechanism of CRE is the production of carbapenemases, which, according to the Ambler classification, are grouped into Class A (KPC), Class B (metallo-β-lactamases, such as NDM and VIM) and Class D (oxacillinases, such as OXA-23).^12^ These enzymes are primarily disseminated through horizontal gene transfer, mediated by mobile genetic elements, such as plasmids and transposons, which facilitates their rapid spread among different bacterial species and clinical environments.^13^ In this context, WGS has become an essential tool for identifying key epidemiological determinants, such as resistance genes, sequence types (STs) and clonal complexes, as well as for analysing transmission pathways at both local and global levels.^1,14^

In Peru, the expansion of carbapenem-resistant Enterobacterales and non-fermenting bacilli poses a growing threat, driven by high-risk clones that harbour carbapenemase genes. In *K. pneumoniae*, a shift has been observed from the *bla*_KPC-2_ gene to the predominance of *bla*_NDM-1_, mobilized by elements, such as Tn125 and conjugative plasmids, with a predominant distribution among lineages ST147, ST45, ST11, and, more recently, ST348.^15–17^ In *P. aeruginosa*, high-risk clones ST357 and ST111 concentrate the *bla*_IMP-93_, *bla*_IMP-18_ and *bla*_VIM-2_ genes, suggesting their adaptation and possible endemicity in the capital of Peru.^18^ In *Escherichia coli*, although less frequent, the first Peruvian strain producing *bla*_NDM-1_ has been documented, belonging to lineage ST155, with a broad resistome against multiple antimicrobial classes.^19^ In contrast, the epidemiological landscape of carbapenem-resistant *A. baumannii* in the country remains poorly defined due to the lack of molecular epidemiological characterization. These findings underscore the need for enhanced molecular surveillance and genomic characterization in local clinical settings. Therefore, the present study aims to contribute to this effort by characterizing the AMR profile, both phenotypically and genotypically, of clinical isolates of Gram-negative bacteria obtained from patients in a referral hospital in Lima, Peru.

## Material and methods

### Study design and collection of clinical data

A cross-sectional study was conducted at Hospital Nacional Arzobispo Loayza, a major national referral centre. Bacterial isolates were collected from hospitalized patients between October 2021 and January 2022. For each isolate, clinical data were retrieved from medical records, including sex, age, source and type of culture, as well as biochemical and haematological laboratory results. Classification of the cases into ‘suspected infection’ and ‘suspected colonization’ was based on clinical and microbiological criteria. This evaluation considered the presence of signs or symptoms consistent with infection, a positive culture result and the attending physician's clinical judgment. Colonization was defined as the detection of a microorganism in the absence of clinical manifestations of infection.

### Microbiology

Bacteria were identified using MALDI-TOF MS, with the MICROFLEX LT/SH system (Bruker Corporation). The collected isolates were subsequently reactivated, and antimicrobial susceptibility testing was performed retrospectively by disk diffusion, following the CLSI M100-2024 standard, using breakpoints to define susceptibility, resistance and intermediate categories, as per the criteria for Enterobacteriaceae and *A. baumannii*. Isolates were considered MDR or XDR if they displayed phenotypic resistance to three or more antibiotic classes.^20^ Carbapenem-resistant isolates were further tested for carbapenemase production using the RESIST-4 O.K.N.V. immunochromatographic assay (OXA-48, KPC, NDM and VIM) (Coris BioConcept, Gembloux, Belgium).

### DNA extraction and whole-genome sequencing

Genomic DNA was extracted using the PureLink™ Genomic DNA Mini Kit (Invitrogen, Cat. No. K182002), following the manufacturer's instructions. Of the 159 isolates included in the study, 78 were subjected to PCR for the detection of carbapenemase genes, including *bla*_NDM_, *bla*_KPC_, *bla*_VIM_ and *bla*_IMP_. A random subset of 47 isolates from the total collection was selected for whole-genome sequencing, irrespective of bacterial species or resistance profile. The number of isolates sequenced was determined by the sequencing capacity available for the study. Genomic libraries were prepared using the Nextera XT DNA Library Preparation Kit (Illumina, San Diego, CA, USA) and indexed with the Nextera XT Index Kit v2. Sequencing was performed on the Illumina MiSeq platform, generating paired-end reads of 250 bp × 2.

### Bioinformatic analysis

Raw reads were assessed with FastQC v0.11.5, and low-quality sequences and adapters were trimmed with Trimmomatic v0.38,^21^ applying a strict Q-score threshold to discard low-quality bases and ensure high-fidelity data. *De novo* assembly was performed using SPAdes v4.2^22^ and, additionally, A5-miseq v20160825 with default parameters. To ensure genome completeness, verify the expected read depth, and detect any potential cross-contamination, the post-assembly quality control was conducted using QUAST. This included the evaluation of fragmentation metrics, such as the number of contigs, the N50 statistic and the total genome length against reference parameters. Molecular typing was performed using the MLST scheme available on pubMLST, for *A. baumannii*, the Oxford MLST scheme was used. AMR genes were identified using ABRicate, which utilized the ResFinder v2.4.0^23^ and CARD v4.0.0^24^ databases, applying thresholds of ≥90 identity and ≥60% alignment coverage.

### Statistical analysis

Categorical variables were described using absolute and relative frequencies. Statistical analysis was performed at a 95% confidence level using STATA v18 (StataCorp, College Station, TX, USA) and R v3.5.2.

## Results

### Patient characteristics

A total of 159 patients were included, with 66.7% being male. The median age was 54 years [interquartile range (IQR): 36–69], with the most represented age groups being those aged 60 years or older (34%) and 50–59 years (22.6%). Clinical evidence of infection was present in 72.3% of patients, and 69.8% of cases were classified as nosocomial in origin. In 27.7% of cases, the origin of infection could not be determined. The most frequent infections were pneumonia (49.1%), urinary tract infections (8.8%) and bacteraemia (8.2%). Most patients (66%) were hospitalized in the ICU, while at admission, 54.7% had already received antibiotic treatment. Regarding comorbidities, 12.6% of patients had some degree of immunosuppression, and 10.1% had cognitive impairment. The median C-reactive protein (CRP) level was 12 mg/L (IQR: 7–23), and 48.4% presented levels compatible with moderate inflammation (10–40 mg/L). The leucocyte count showed a median of 10 560/mm^3^ (IQR: 7800–14 180); 46.5% of patients presented with leucocytosis (>11 000/mm^3^), and 6.9% with leucopenia (<4000/mm^3^) (Table 1).

**Table 1.** Clinical characteristics and laboratory findings of patients included in the study (*N* = 159)

Patient clinical data	Total (*N* = 159)
	*n* (%)
Sex	
Male	106 (66.7)
Female	53 (33.3)
Age (years)	
16–29	32 (20.1)
30–39	18 (11.3)
40–49	19 (11.9)
50–59	36 (22.6)
60+	54 (34)
Body mass index (BMI)	
Underweight (<18.5)	1 (0.63)
Normal weight (18.5–24.9)	49 (30.82)
Overweight (25–29.9)	78 (49.06)
Obesity (≥30)	31 (19.5)
Presence of infection	
Yes	115 (72.3)
No	44 (27.7)
Nosocomial infection	
Yes	111 (69.8)
No	4 (2.5)
Undetermined	44 (27.7)
Type of infection	
Pneumonia	78 (49.1)
Urinary tract infection	14 (8.8)
Bacteraemia	13 (8.2)
Skin and soft tissue infection	7 (4.4)
Bone infection	1 (0.6)
Intra-abdominal infection	2 (1.3)
Undetermined	44 (27.7)
Specimen type	
Bronchial	100 (62.9)
Urine	31 (19.5)
Blood	13 (8.2)
Wound	7 (4.4)
Tissue	3 (1.9)
Bone	2 (1.3)
Bile	2 (1.3)
Ascitic fluid	1 (0.6)
Hospital ward	
Intensive care unit	105 (66.0)
Internal medicine	22 (13.8)
Surgery	21 (13.2)
Emergency	9 (5.7)
Paediatrics	2 (1.3)
Antibiotic use at admission	
Yes	87 (54.7)
No	72 (45.3)
Immunosuppression	
No	139 (87.4)
Yes	20 (12.6)
Cognitive impairment	
Yes	16 (10.1)
No	143 (89.9)
C-reactive protein (CRP)	
Normal or low risk (<10 mg/L)	50 (31.4)
Moderate inflammation (10–40 mg/L)	77 (48.4)
Severe inflammation (>40 mg/L)	2 (1.3)
Undetermined	30 (18.9)
Leucocyte count	
Leucopenia (<4000/mm^3^)	11 (6.9)
Normal (4000–11 000/mm^3^)	68 (42.8)
Leucocytosis (>11 000/mm^3^)	74 (46.5)
Undetermined	6 (3.8)

*N*, total number of cases; *n*, number of cases in each category; %, percentage.

### Phenotypic and molecular resistance profiles

Of the 159 isolates evaluated, 97.5% exhibited an XDR phenotype and 2.5% a MDR phenotype. XDR resistance was universal (100%) in *A. baumannii*, *P. aeruginosa*, *E. coli*, *Providencia rettgeri*, *Proteus mirabilis* and *Acinetobacter haemolyticus* and high in *K. pneumoniae* (91.8%). Among β-lactam/β-lactamase inhibitor combinations, resistance to piperacillin/tazobactam was 97.5%, with rates ≥93.8% in *P. aeruginosa*, *K. pneumoniae* and *E. coli*. Resistance to ceftazidime/avibactam reached 91.2%, being 100% in *K. pneumoniae*, *E. coli*, *P. rettgeri*, *P. mirabilis* and *A. haemolyticus.* Similar patterns were observed with aztreonam (90.6%), although with lower activity against *P. aeruginosa* (79.6%) and no detectable activity against *P. rettgeri* and *A. haemolyticus*. Resistance to ceftriaxone and ceftazidime was particularly high in *K. pneumoniae* (100% and 91.8%, respectively) and lower against *P. aeruginosa* (55.1% and 69.4%). Regarding carbapenems, resistance rates were close to 100%: imipenem (99.4%), meropenem (98.7%) and ertapenem (98.3%). All species showed complete resistance, except for *K. pneumoniae* and *P. aeruginosa*, where meropenem showed slightly lower values (97.9%). Ciprofloxacin resistance was 98.1% overall, with values of 100% in most species, except in *A. baumannii* (97.5%). In contrast, amikacin and tigecycline retained better activity, with resistance to amikacin at 20.1%, low in *K. pneumoniae* (12.2%) and absent in *E. coli* and *P. rettgeri*. Tigecycline showed an overall resistance of 12.1%, with low rates in *A. baumannii* (6.1%) and *K. pneumoniae* (16.3%) and no resistance in *E. coli*, *P. rettgeri* and *A. haemolyticus*. In molecular analysis by PCR (*n* = 78), the NDM gene was identified in 69.2% of isolates, especially in *K. pneumoniae* (91.7%), *P. rettgeri* (100%) and *P. mirabilis* (100%). IMP genes (14.1%), KPC (5.1%), VIM (3.9%) and VIM/IMP combinations (7.7%) were also detected, mainly in *P. aeruginosa* and *Enterobacter cloacae* (Table 2).

**Table 2.** Antimicrobial resistance profiles of Gram-negative clinical isolates by bacterial species

Antimicrobial Resistance results	Total	*Acinetobacter baumannii* (*n* = 49) (%)	*Pseudomonas aeruginosa* (*n* = 49) (%)	*Klebsiella pneumoniae* (*n* = 49) (%)	*Enterobacter cloacae* (*n* = 3) (%)	*Escherichia coli* (*n* = 3) (%)	*Providencia rettgeri* (*n* = 4) (%)	*Proteus mirabilis* (*n* = 1) (%)	*Acinetobacter haemolyticus* (*n* = 1) (%)
β-Lactam/β-lactamase inhibitor combinations									
Ampicillin/sulbactam (*n* = 110)	85 (77.7)	25 (51.0)	—	49 (100.0)	3 (100.0)	3 (100.0)	4 (100.0)	1 (100.0)	0 (0.0)
Piperacillin/tazobactam (*n* = 158)	154 (97.5)	49 (100.0)	45 (93.8)	48 (98.0)	3 (100.0)	3 (100.0)	4 (100.0)	1 (100.0)	1 (100.0)
Ceftazidime/avibactam (*n* = 159)	145 (91.2)	39 (79.6)	45 (91.8)	49 (100.0)	3 (100.0)	3 (100.0)	4 (100.0)	1 (100.0)	1 (100.0)
Ceftazidime (*n* = 109)	89 (81.7)	—	34 (69.4)	45 (91.8)	3 (100.0)	3 (100.0)	3 (75.0)	1 (100.0)	—
Ceftriaxone (*n *= 109)	96 (78.9)	—	27 (55.1)	49 (100.0)	3 (100.0)	3 (100.0)	3 (75.0)	1 (100.0)	—
Cefepime (*n* = 156)	129 (82.7)	26 (55.3)	45 (91.8)	48 (97.5)	3 (100.0)	3 (100.0)	4 (100.0)	—	0 (0.0)
Aztreonam (*n* = 159)	144 (90.6)	49 (100.0)	39 (79.6)	48 (98.0)	3 (100.0)	3 (100.0)	2 (50.0)	0 (0.0)	0 (0.0)
									
Carbapenems									
Ertapenem (*n* = 60)	59 (98.3)	—	—	49 (100.0)	3 (100.0)	3 (100.0)	3 (75.0)	1 (100.0)	—
Imipenem (*n* = 155)	154 (99.4)	49 (100.0)	1 (100.0)	47 (100.0)	3 (100.0)	3 (100.0)	3 (75.0)	1 (100.0)	1 (100.0)
Meropenem (*n* = 159)	157 (98.7)	49 (100.0)	49 (100.0)	48 (98.0)	3 (100.0)	3 (100.0)	3 (75.0)	1 (100.0)	1 (100.0)
									
Aminoglycosides									
Amikacin (*n* = 159)	32 (20.1)	11 (22.5)	13 (26.5)	6 (12.2)	0 (0.0)	0 (0.0)	1 (25.0)	1 (100.0)	0 (0.0)
Fluoroquinolones									
Ciprofloxacin (*n* = 159)	156 (98.1)	48 (97.5)	1 (100.0)	49 (100.0)	3 (100.0)	3 (100.0)	4 (100.0)	1 (100.0)	1 (100.0)
									
Glycylcyclines									
Tigecycline (*n* = 107)	13 (12.2)	3 (6.1)	—	8 (16.3)	0 (0.0)	0 (0.0)	2 (100.0)	—	0 (0.0)
									
Resistance profile (*n* = 159)									
XDR	155 (97.5)	49 (100.0)	49 (100.0)	45 (91.8)	3 (100.0)	3 (100.0)	4 (100.0)	1 (100.0)	1 (100.0)
MDR	4 (2.5)	0 (0.0)	0 (0.0)	4 (8.2)	0 (0.0)	0 (0.0)	0 (0.0)	0 (0.0)	0 (0.0)
									
Carbapenemase PCR results (*n* = 78)									
IMP	11 (14.1)	0 (0.0)	9 (50.0)	0 (0.0)	2 (66.7)	0 (0.0)	0 (0.0)	0 (0.0)	0 (0.0)
KPC	4 (5.1)	0 (0.0)	0 (0.0)	4 (8.3)	0 (0.0)	0 (0.0)	0 (0.0)	0 (0.0)	0 (0.0)
NDM	54 (69.2)	1 (100.0)	0 (0.0)	44 (91.7)	1 (33.3)	3 (100.0)	3 (100.0)	1 (100.0)	1 (100.0)
VIM	3 (3.9)	0 (0.0)	3 (16.7)	0 (0.0)	0 (0.0)	0 (0.0)	0 (0.0)	0 (0.0)	0 (0.0)
VIM/IMP	6 (7.7)	0 (0.0)	6 (33.3)	0 (0.0)	0 (0.0)	0 (0.0)	0 (0.0)	0 (0.0)	0 (0.0)

Note: *n* indicates the total number of isolates included or tested in the corresponding analysis. For antimicrobial susceptibility results, the *n* shown next to each antimicrobial agent represents the total number of isolates tested, whereas values in the cells are presented as the number of resistant isolates followed by the corresponding percentage. For carbapenemase PCR results, *n* represents the number of isolates tested by PCR. Percentages were calculated using the corresponding number of isolates tested as the denominator.

### Genomic characterization

A total of 47 genomes underwent *de novo* assembly and subsequent quality assessment. The assembled genomes demonstrated robust contiguity and high quality, validating their suitability for downstream resistome and epidemiological profiling. The median number of contigs per assembly was 170 (IQR: 130–241). Furthermore, the N50 quality parameter, an indicator of assembly contiguity, showed a median of 79 669 bp (IQR: 53 852–146 326 bp). The total sequence length across the clinical isolates ranged between 3.9 and 7.2 Mbp, maintaining GC contents characteristic of their respective taxa, which ranged from 38.95% to 66.38%. These post-assembly metrics confirmed the absence of significant contamination and validated the overall sequencing depth and coverage for the analysed Gram-negative bacilli.

In *A. baumannii* (*n* = 14), four distinct STs were identified: ST1163 (*n* = 6), ST1079 (*n* = 4), ST2 (*n* = 3) and ST25 (*n* = 1). ST1163 showed a high load of aminoglycoside resistance genes—*aac(6′)-Ian*, *aph(3′)-Ia*, *aph(6)-Id* and *strA*—and the carbapenemase gene *bla*_OXA-23_ in all isolates. ST2 shared a similar pattern, with the additional presence of the armA gene in one isolate. In contrast, ST1079 was characterized by a more limited profile, expressing only *aph(6)-Id* and *bla*_OXA-23_, while ST25 exhibited a broader repertoire, including aminoglycoside resistance genes and Class D β-lactamases. In *K. pneumoniae* (*n* = 20), greater clonal diversity was found with five different STs: ST13 (*n* = 8), ST1128 (*n* = 4), ST15 (*n* = 3), ST147 (*n* = 2) and ST45 (*n* = 1). ST13 was the most frequent and showed a high accumulation of resistance genes, including *aac(6′)-Ib-cr*, *aph(3′)-Ia*, *aph(6)-Id*, *strA*, *bla*_CTX-M-15_, *bla*_KPC-2_, *sul1*, *sul2*, *oqxA/B*, *qnrE1*, *mph(A)*, *fosA* and *fosA6*. A very similar profile was observed in clones ST15, ST1128 and ST147, suggesting possible clonal dissemination of multidrug-resistant variants within the hospital setting. In *P. aeruginosa* (*n* = 9), ST357 was the most prevalent (*n* = 3) and was consistently associated with aminoglycoside resistance genes, such as *aph(3″)-Ib*, *aph(3′)-IIb*, *aph(6)-Id* and *strA*, in addition to carbapenemase genes *bla*_IMP-16_ and *bla*_VIM-2_. Other clones, including ST111 (*n* = 2), ST1230, ST235, ST274 and ST179, also expressed variable combinations of resistance genes, particularly *mph(A)* and β-lactamase genes in ST235. The two *E. coli* isolates corresponded to ST167 and shared a homogeneous genetic profile, including *aac(6′)-Ib-cr*, *aph(3″)-Ib*, *aph(6)-Id*, *catB4*, *sul1*, *sul2*, *oqxA/B*, *qnrE1*, *fosA* and *fosA6*, confirming the association of this clone with a multidrug-resistant phenotype. Finally, in *E. cloacae* (*n* = 1, ST78), the *bla*_IMP-16_, *bla*_NDM-5_, *catB4*, *sul1* and *fosA* genes were detected, consistent with a resistance profile to multiple antimicrobial classes (Figure 1).

**Figure 1.**
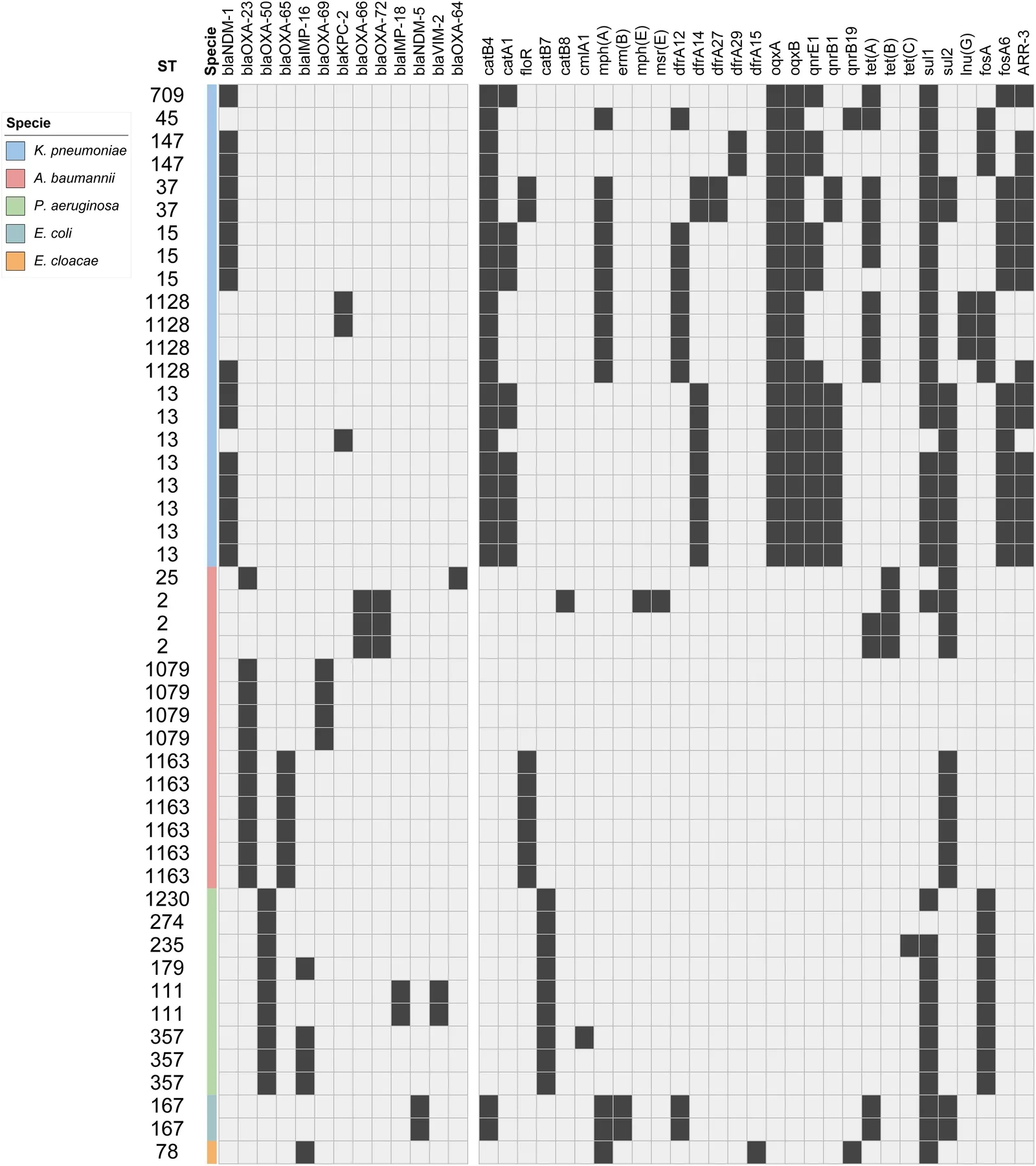
Resistance genes in carbapenem-resistant Gram-negative bacilli: Antimicrobial resistance genes identified in carbapenem-resistant Gram-negative bacilli are presented by bacterial isolate, together with individually annotated resistance genes.

## Discussion

In this single-centre cross-sectional study of 159 carbapenem-resistant Gram-negative bacilli collected between October 2021 and January 2022, 97.5% of isolates were classified as XDR. This high proportion should be interpreted in the context of the study design, which specifically included carbapenem-resistant isolates and therefore does not represent the prevalence of XDR among all Gram-negative isolates or infections at the hospital. In *K. pneumoniae*, *bla*_NDM-1_ and *bla*_KPC-2_ were identified, both associated with carbapenem resistance. Among the carbapenemase genes detected, *bla*_NDM-1_ accounted for 90.5%, whereas *bla*_KPC-2_ accounted for 14.3%, supporting the increasing relevance of NDM-mediated resistance in the Peruvian context.^16,17^

In the sequenced *K. pneumoniae* collection, ST13 and ST15 were notable for the frequent detection of *bla*_NDM-1_. Specifically, 87.5% (7/8) of ST13 isolates carried *bla*_NDM-1_, whereas this gene was detected in all ST15 isolates (3/3, 100%). This pattern is consistent with recent genomic studies from Lima showing the increasing relevance of *bla*_NDM-1_ and its occurrence across diverse *K. pneumoniae* genetic backgrounds, including ST45, ST11, ST147, ST348 and ST15.^16,17^ In addition, NDM-producing ST15 *K. pneumoniae* has been reported in Brazil, supporting the regional relevance of this high-risk lineage.^25^ Rather than indicating local clonal expansion, the detection of *bla*_NDM-1_ in ST13 and ST15 broadens the diversity of genetic backgrounds associated with carbapenem resistance in Peru. Longitudinal surveillance combined with high-resolution genomic analysis would be required to determine whether the repeated detection of these STs reflects persistence or local transmission.

In this study, *A. baumannii* represented 30.8% (49/159) of isolates and showed an XDR profile, with resistance rates exceeding 95% to ciprofloxacin, imipenem and meropenem. These antibiotics play a key role in the management of severe Gram-negative bacillus infections, especially in hospital settings,^26^ while increased resistance of *Acinetobacter* spp. to fluoroquinolones has also been reported in outpatient care.^27^ At the genomic level, all 14 sequenced *A. baumannii* isolates carried *bla*_OXA-23_, an acquired carbapenemase widely associated with carbapenem resistance in this species and among the most widely disseminated carbapenemases in Latin America.^28^ Molecular studies from tertiary hospitals in Lima have also documented carbapenem resistance and OXA-type carbapenemases, including OXA-23.^29^ More recent genomic surveillance in Lima identified *bla*_OXA-23_ in XDR *A. baumannii* lineages *ST79* and *ST1079*.^30^ Together, these findings support the regional importance of OXA-23-mediated resistance; however, the limited number of sequenced isolates and short study period do not allow conclusions about the predominance or expansion of specific lineages.

A noteworthy finding was the detection of *floR*, which is associated with resistance to florfenicol, an antimicrobial agent used primarily in veterinary medicine.^31^ Previous studies have reported *floR* in human clinical isolates and mobile genetic elements, including transferable plasmids in *K. pneumoniae*.^32^ Because plasmid structure and the genomic context of *floR* were not characterized in this study, its origin and potential route of acquisition cannot be determined. Its detection supports the value of One Health genomic surveillance to investigate possible links among human, animal and environmental reservoirs. Multiple aminoglycoside resistance determinants were also identified, including *aph(6)-Id*, *aac(6′)-Ib-cr* and *strA*; *strA* was present in 83.3% of isolates, whereas *aac(6′)-Ian* was detected in all samples. These genes contribute to resistance against clinically relevant aminoglycosides, although their antimicrobial spectra differ. In particular, *aac(6′)-Ib-cr* can also reduce susceptibility to certain fluoroquinolones,^33^ whereas *aac(6′)-Ian* has been associated primarily with resistance to aminoglycosides, such as amikacin, kanamycin, tobramycin and netilmicin.^34^

In Peru, ST111 (44.0%) and ST357 (38.3%) have been reported as predominant high-risk *P. aeruginosa* lineages among carbapenemase-producing isolates. Previous studies described *bla*_IMP-18_/*bla*_VIM-2_ in ST111 and *bla*_IMP-93_ in ST357.^18^ In our study, ST357 (*n* = 3) and ST111 (*n* = 2) were the most frequently identified STs among the nine sequenced *P. aeruginosa* isolates. Figure 1 shows *bla*_IMP-18_ and *bla*_VIM-2_ in ST111, whereas *bla*_IMP-16_ was identified in ST357. In Colombia, ST235 has been associated with multidrug resistance and *bla*_KPC-2_ in hospital settings.^35^ A report from Spain also described carbapenem-resistant *P. aeruginosa* ST179 carrying *bla*_IMP-16_ and KPC variants in patients linked to Peru.^36^ ST111 carrying *bla*_IMP-18_ and *bla*_VIM-2_ has been documented in Central America.^37^ The small number of sequenced isolates does not allow conclusions about the predominance, endemicity or sustained local expansion of these lineages.

This study has several limitations. First, it was conducted at a single referral hospital over a 4-month period, which limits the generalizability of the findings and precludes conclusions regarding sustained transmission, clonal expansion or endemicity. Second, the study specifically included carbapenem-resistant Gram-negative bacilli; therefore, the high proportion of XDR isolates should not be interpreted as representative of all Gram-negative isolates or infections at the hospital. Third, PCR was performed on 78 isolates and WGS on a random subset of 47; consequently, the observed distribution of STs and resistance determinants may not fully represent the diversity of the complete collection or detect less frequent or emerging lineages. In addition, MLST and resistome profiling alone do not provide sufficient resolution to demonstrate recent clonal transmission, for which high-resolution phylogenomic analysis would be required. Finally, plasmid reconstruction and characterization of the genetic context surrounding resistance genes were not performed, limiting conclusions regarding their potential horizontal dissemination.

Despite these limitations, this study provides a 4-month genomic snapshot of carbapenem-resistant Gram-negative bacilli recovered at a referral hospital in Lima, Peru. Among the sequenced isolates, the findings included *bla*_NDM-1_ in *K. pneumoniae* ST13 and ST15, *bla*_OXA-23_ in all 14 *A. baumannii* isolates and *bla*_IMP-18_/*bla*_VIM-2_ in *P. aeruginosa* ST111 and *bla*_IMP-16_ in ST357. These results illustrate the diversity of resistance determinants in this selected collection and support continued molecular surveillance, antimicrobial stewardship and infection-prevention measures. They do not establish hospital-wide prevalence, transmission or clonal expansion.

## Ethical and institutional approval

The study protocol was reviewed and approved by the Institutional Research Committee of Hospital Nacional Arzobispo Loayza (Evaluation Report No. 021-CII-HNAL/2024, 12 February 2025) and by the Office for Teaching and Research Support (Approval No. 023-OADEI-HNAL/2025, 14 February 2025). Final institutional authorization for the execution of the study was granted by the Hospital Directorate through Letter No. 049-DG-HNAL/2025, dated 26 February 2025.

## Data Availability

The whole-genome sequencing data, including assembled genomes generated during this study, are publicly available in the NCBI repository under the BioProject accession number PRJNA1503038.
